# Face Perception and Narcissism: Variations of Event-Related Potential Components (P1 & N170) with Admiration and Rivalry

**DOI:** 10.3758/s13415-020-00818-0

**Published:** 2020-08-14

**Authors:** Markus Mück, Katharina Ohmann, Sebastian Dummel, André Mattes, Ulrike Thesing, Jutta Stahl

**Affiliations:** Department of Individual Differences and Psychological Assessment, University of Cologne, Pohligstr 1, 50969 Köln, Germany

**Keywords:** P1, N170, event-related potentials (ERP), narcissism, Narcissistic Admiration and Rivalry Concept (NARC)

## Abstract

Previous studies have demonstrated that highly narcissistic individuals perceive themselves as grandiose and devaluate and sometimes overvalue others. These results are mainly based on behavioural data, but we still know little about the neural correlates underlying, such as perceptional processes. To this end, we investigated event-related potential components (ERP) of visual face processing (P1 and N170) and their variations with narcissism. Participants (N = 59) completed the Narcissistic Admiration and Rivalry Questionnaire and were shown pictures of their own face, a celebrity’s face, and a stranger’s face. Variations of P1 and N170 with Admiration and Rivalry were analysed using multilevel models. Results revealed moderating effects of both narcissism dimensions on the ERP components of interest. Participants with either *high* Admiration or *low* Rivalry scores showed a lower P1 amplitude when viewing their own face compared with when viewing a celebrity’s face. Moreover, the Self-Stranger difference in the N170 component (higher N170 amplitude in the Self condition) was larger for higher Rivalry scores. The findings showed, for the first time, variations of both narcissism dimensions with ERPs of early face processing. We related these effects to processes of attentional selection, an expectancy-driven perception, and the mobilisation of defensive systems. The results demonstrated that by linking self-report instruments to P1 and N170, and possibly to other ERP components, we might better understand self- and other-perception in narcissism.

Today’s concept of narcissism is rooted in ancient mythology. In Ovid’s *Metamorphoses* (1916, pp. 153-155), the author describes how Narcissus falls in love with himself, seeing his face in the silver white water of an unclouded fountain. Nothing can release his eyes from his face, and he falls into deep despair realising that he cannot reach what he sees. Although this narrative—depicting Narcissus’ perception of his face—marks the starting point of our modern understanding of narcissism, to date, we still know little about face perception in narcissism and its underlying psychological and neurophysiological processes.

To date, mainly behavioural studies have suggested that narcissistic people are very fond of their own faces. For example, narcissism, at least in men, was shown to be positively associated with the number of selfies posted on social media platforms (Sorokowski et al., 2015). Furthermore, adolescents high in narcissism rated posted photos of themselves as being more glamorous, more fashionable, and more physically attractive compared with less narcissistic adolescents (Ong et al., 2011). It also was postulated that people high in narcissism use their appearance to signal their actual or desired status (Vazire, Naumann, Rentfrow, & Gosling, 2008) and to gain attention and admiration by others (Sedikides, Gregg, Cisek, & Hart, 2007). The special relationship between highly narcissistic individuals with their appearance and their face, further manifests in the item: “I like looking at myself in the mirror,” which was incorporated into the most widely used narcissism inventory (Narcissistic Personality Inventory; Raskin & Terry, 1988).

Using functional magnetic resonance imaging (fMRI), a recent study demonstrated, however, that while viewing their face, compared with viewing friends’ or strangers’ faces, highly narcissistic men showed more neural activity in the anterior cingulate cortex; the authors interpreted this finding as an indicator of a negative affect during self-relevant processing (Jauk, Benedek, Koschutnig, Kedia & Neubauer, 2017). The inconsistency between the mentioned behavioural data and this neurophysiological finding shows that we still know little about the processes underlying self-perception in narcissism.

Because individuals do not live alone in this world and interact with others daily, other people’s faces also might be important cues in a social context for more narcissistic individuals. The existing literature showed that narcissistic people often dictate the nature of these interactions. For example, Back et al. (2013) postulated that individuals high in narcissism use social encounters to stabilise their self-worth by devaluing their interaction partners. Furthermore, Campbell and Green (2007) emphasised that highly narcissistic individuals use social interactions as an opportunity to be admired. In some instances, individuals high in narcissism even overvalue other people—for example, because of their high social reputation—when this serves the stabilisation of their own grandiosity (Campbell & Green, 2007). Such social interactions usually begin with the perception of another person’s face (Ofan, Rubin, & Amodio, 2011). Thus, neurophysiological correlates of face processing also could be informative for narcissism-related variations in the perception of other people.

To assess narcissism-related variations in face processing on a neural level, we investigated neurophysiological correlates of face perception using event-related potentials (ERPs). We opted for this approach, because the use of ERPs is beneficial to identify variations in the temporal dynamics of neural processes—an important aspect in social cognitive and affective neuroscience (Amodio, Bartholow, & Ito, 2013).

Considering temporal dynamics seems to be especially important when studying narcissism. With a priming paradigm, Horvath and Morf (2009) showed that, at an early stage of information processing, highly narcissistic individuals were hypersensitive to words representing worthlessness but, at a later stage, automatically and successfully avoid experiencing worthlessness. They argued that the uncertainty surrounding the existence of implicit experiences of worthlessness in narcissism had been caused by the negligence of temporal dynamics in most study designs.

The ERP approach, however, enabled us to study different processing stages with a high temporal resolution. Most importantly, it allowed us to investigate processes immediately after stimulus onset. Thus, this approach provided insights into rather automatic responses that are difficult or impossible to detect with other methods often applied in the research on narcissism, such as self-report (Tamborski & Brown, 2011), clinical observations (Afek, 2018) or fMRI (Jauk et al., 2017). Specifically, the current study used two well-studied components of the ERP associated with face processing: P1 and N170.

## Face Processing Components

First, we assessed the P1 component, which peaks between 80 and 130 ms post-stimulus (Hillyard & Anllo-Vento, 1998) and originates both from the dorsal extrastriate cortex and from the fusiform gyrus (Di Russo, Martínez, Sereno, Pitzalis, & Hillyard, 2001). Although this early component varies with low-level information of visual stimuli (Rossion & Jacques, 2008), P1 has also been found to correlate with conscious perception: It was shown that the P1 amplitude is enhanced when participants consciously perceive stimuli compared with when they do not consciously perceive them (Mathewson, Gratton, Fabiani, Beck, & Ro, 2009; Roeber, Trujillo-Barreto, Hermann, O’Shea, & Schroger, 2008; Kornmeier & Bach, 2006; Pins, 2003). Railo, Koivisto, and Revonsuo (2011) postulated, however, that the P1 reflects a preconscious attentional selection process which controls the visual content that enters into consciousness. This sensory gain control mechanism is manifested either as attentional suppression or as attentional facilitation, occurring at an early stage of information processing, before the stimulus is fully identified and recognised (Hillyard, Vogel, & Luck 1998). Interestingly, the emotional significance of stimuli affects early attention-modulating processes (Vuilleumier, 2005). It is argued that via neuronal projections to the sensory cortices, the amygdala can influence and reinforce the perception of emotional and intrinsically salient events—a process that is termed emotional attention (Vuilleumier, 2005). This was demonstrated in several studies investigating the association between negatively valenced stimuli and the P1 amplitude. For example, enhanced P1 amplitudes were found in socially phobic patients while seeing faces (Kolassa, Kolassa, Musial, & Miltner, 2007) or angry faces (Mueller et al., 2008), in spider phobics while viewing spiders (Michalowski et al., 2009), in participants seeing fearful faces (Batty & Taylor, 2003), and in response to negatively compared with positively valenced stimuli (Smith, Cacioppo, Larsen, & Chartrand, 2003). To substantiate the association between the emotional valence of stimuli with the P1 amplitude, Rotshtein et al. (2010) showed that patients with amygdala damage did not show an increased P1 in response to fearful faces compared with neutral ones. Smith et al. (2003) highlighted that the P1 amplification to negative stimuli pointed to a mechanism at a very early stage of information processing, which seemed to ascribe valence to sensory input, leading to a preference for negative over positive stimuli in the process of perception. The authors argued that, from an evolutionary perspective, this mechanism is essential for reacting quickly and appropriately to (life-) threatening events.

The second ERP component that we focused on in the current research was the N170 component. This component is discussed to reflect higher-order face-sensitive brain processes—the structural encoding of faces—as its amplitude is higher for faces compared to other non-face objects (Rossion & Jacques, 2011). N170 shows its local maximum at posterior electrode sites, i.e., above the visual cortical areas, and peaks around 170 ms after presentation of the stimulus (Bentin, Allison, Puce, Perez, & McCarthy, 1996; Eimer, 2000; Rossion & Jacques, 2011; Eimer, 2011). Even though the perceptual processes underlying the N170 can also be recruited for other non-face visual stimuli of expertise (for example birds and dogs [Tanaka & Curran, 2001], or fingerprints [Busey & Vanderkolk, 2005]), this component, in particular, seems to reflect the higher-level process of perceiving a visual stimulus as a face (see Rossion & Jacques, 2011). With regard to the current study, the so-called self-effect of N170 is essential (Keyes, Brady, Reilly, & Foxe, 2010); this describes larger N170 amplitudes when participants see their own face compared with when they see the face of a friend or a stranger (defined by an N170 difference). Given the self-importance, narcissistic people feel for themselves (Krizan & Herlache, 2018), we assumed that this N170 self-effect might even be enhanced in narcissism. Furthermore, previous research has demonstrated that the social significance of other faces leads to an increase in the N170 amplitude. Participants viewing a member ostensibly of their own social group—arbitrarily assigned by the experimenters—showed a larger N170 compared with ostensible out-group faces, suggesting a motivational preference in the encoding of faces of in-group members (Ratner & Amodio, 2013). Additionally, increased N170 amplitudes also were demonstrated for white participants while observing black faces compared with white faces, but only if the participants were frightened of showing racial prejudice (Ofan, Rubin, & Amodio, 2014) or had implicit pro-white attitudes (Ofan et al., 2011). Moreover, it was shown that social conformity regarding attractiveness ratings led to smaller N170 amplitudes (Schnuerch, Koppehele-Gossel, & Gibbons, 2015). Given the importance of interpersonal self-regulation for narcissistic grandiosity (Campbell & Green, 2007), the illustrated effects, concerning the interplay between social significance and the N170 amplitude, might be moderated by narcissism.

## Narcissistic Admiration and Rivalry Concept

The current literature provides a variety of concepts about narcissism, each with different or slightly different foci (Pincus & Lukowitsky, 2010). To our knowledge, P1 and N170 were not used to investigate variations with any narcissism concept in previous studies. To explore variations of these ERP components with narcissism, we focused on the Narcissistic Admiration and Rivalry Concept (NARC; Back et al., 2013). The NARC, a concept allowing a dimensional investigation of narcissism, postulates two distinct pathways according to which narcissistic people can maintain their grandiose self: Admiration and Rivalry. Both pathways incorporate affective, motivational, cognitive and behavioural processes. Admiration is associated with a strategy of maintaining grandiosity by attaining the admiration of other people (assertive self-enhancement). This strategy is associated with the aim to present one’s uniqueness and specialness, with fantasies about one’s grandiosity and with charming behaviour that can lead to positive social outcomes. These positive social experiences, in turn, drive the grandiose self and further reinforce the assertive self-enhancement strategy. Rivalry, on the other hand, reflects the process in which one’s grandiosity is defended from attacks by other people (antagonistic self-protection). This self-defence is linked with the striving to prove superiority, with the cognitive strategy of devaluing others and with aggressive behaviour. This self-protection mechanism leads to negative social outcomes which, in turn, stabilises the negative view of others and ultimately results in a strengthening of this antagonistic self-protection strategy.

Thus, the NARC postulates that the Admiration dimension reflects the tendency of perceiving oneself as (more or less) grandiose while the Rivalry dimension reflects the tendency of perceiving others (more or less) as inferior. We assumed that these differences in perception might also be related to variations in P1 and N170 while viewing oneself or other people. By describing these differences in perception at a psychological level, the NARC seemed to be a suitable model for investigating variations in face processing at a neural level.

## Objective of the Present Research

We showed participants three different kinds of faces (the participant’s own face, the face of a stranger, and a celebrity’s face) and explored variations in P1 and N170 that can be explained by variations in Admiration and Rivalry. First, we investigated the general effects of Face Type on P1 and N170 and tried to replicate the so-called self-effect on N170 (Keyes et al., 2010). Second, we tested whether both Admiration and Rivalry moderated the effect of Face Type on P1 and N170. Based on the outlined theoretical considerations, one’s own face should be an important stimulus for people with high Admiration: Viewing one’s own face poses an opportunity to feel grandiose. A stranger´s face should be an important stimulus for people high in Rivalry: Viewing a stranger’s face poses an opportunity to devaluate another person. Thus, the importance of the respective stimulus should lead to an intensified face processing reflected in variations in the P1 and the N170 component. We, therefore, investigated if P1 and N170 varied for participants high in Admiration while viewing one’s own face compared with viewing a celebrity’s and a stranger’s face, and if P1 and N170 varied for participants high in Rivalry while viewing a stranger’s face. Furthermore, we assumed variations of either Admiration or Rivalry with P1 and N170 when viewing a celebrity’s face since Campbell and Green (2007) pointed out that the affiliation with people of high social status, also, poses an opportunity for highly narcissistic individuals to stabilize their grandiosity.

We supposed that both ERP components vary with Admiration and Rivalry. However, given the vast and complex P1 and N170 literature, we could not draw any directed hypotheses on how the two personality traits might affect the ERP components. The study was, above all, explorative; we aimed at generating data providing first insights into a better understanding of early face processing in narcissism.

## Methods

### Participants

We recruited 61 right-handed participants studying at the University of Cologne who received course credit for participation. Two participants had to be removed from this sample because of technical problems, resulting in a final sample of 59 participants (42 females, 17 males, no one identified as diverse; mean age = 25.45 years, *SD* = 6.22). All participants reported that they had never suffered from a neurological illness and had either normal or corrected-to-normal vision. The study was approved by the ethics committee of the German Psychological Association. Participants gave written consent.

### Psychometric assessment

Narcissism was measured using the 18-Items Narcissistic Admiration and Rivalry Questionnaire (NARQ, Back et al., 2013). The NARQ measures the affective-motivational, cognitive, and behavioural aspects of both facets of narcissism (Admiration and Rivalry). The Admiration scale incorporates three subscales, including Grandiose Fantasies (cognitive aspect), Striving for Uniqueness (affective-motivational aspect), and Charmingness (behavioural aspect). The Rivalry scale consists of another three subscales, including Devaluation (cognitive aspect), Striving for Supremacy (affective-motivational aspect), and Aggressiveness (behavioural aspect). Participants respond on a 6-point Likert scale ranging from 1 = *not agree at all* to 6 = *agree completely*. The internal consistency of scores on the Admiration subscale was α = 0.76, the internal consistency for Rivalry scores was α = 0.82. The sample’s mean and standard deviation for Admiration were 3.02 ± 0.62 (range: 1.67 to 4.33, centred range: −1.35 to 1.32) and for Rivalry 1.92 ± 0.71 (range: 1.00 to 4.00, centred range: −0.92 to 2.08).

### Materials

During the experimental task, three categories of photos (all matching the participant’s sex) were presented. First, the participants saw photos of their own face (Self condition). This was managed by photographing all participants before the experimental task in front of a white wall. These photos were matched to the other stimuli presented during the experimental task (i.e., pictures of celebrities and strangers; see below). This was done by transforming them into black and white pictures and by adjusting them systematically with regard to picture detail, contrast, lightness, and size. Second, in the Celebrity condition, either a photo of Brad Pitt (shown to male participants) or a photo of Angelina Jolie (shown to female participants) was presented on the screen. These photos had been edited in the same way as the photos for the Self condition. Third, in the Stranger condition, a stranger’s face of the same sex as the participant was presented on the screen. The photo of the female stranger’s face was taken from a stimulus set of female faces that was used in a prior study, and this photo had been rated as moderately attractive (Ohmann, Stahl, Mussweiler, & Kedia, 2016). Similar to Ohmann et al. (2016), we generated a second stimulus set containing 91 male faces that were pretested for attractiveness in a separate male sample (*N* = 13; mean age = 23.54 years). One of these photos that also had been rated as moderately attractive was presented to our male participants.

Additionally, 90 stimuli showing each a different stranger´s face were used as filler items. These photos originated from one of the above-mentioned stimulus sets and were again matched to the participant´s sex. They were implemented to counteract vigilance decrement that can be caused by insufficient workload (Manly, Robertson, Galloway, & Hawkins, 1999). The participants had to rate the social competencies of the person who was presented on the screen (see below); including these additional 90 photos of stranger faces, they did not only rate three stimuli but a variety of different stimuli during the experiment.

### Experimental task and procedure

After editing each participant’s photo for the Self condition, the participants were prepared for the experimental task (programmed in E-Prime; Psychology Software Tools, Pittsburgh, PA). Participants were placed in front of a computer screen with their head on a chin rest (60-cm distance to the screen) to reduce unwanted movements during the task. The experimental task was divided into 3 blocks of 120 trials. Within each block, pictures of one’s own, the celebrity’s, and the stranger’s face were each presented 30 times in random order and interspersed with 30 different stranger faces. Blocks were separated by a 2-minute break. Every individual trial started with the presentation of a face. Then, 800 ms after stimulus onset, an analogue scale appeared on the screen, and the participants had to rate the perceived social competencies of the person presented. This instruction was used to keep the participants focused on the faces during the experiment. Every stimulus remained on the screen until the rating was finished. After stimulus offset, a blank screen occurred for 500 ms. In total, it took participants approximately 15 minutes to complete the task. Following the EEG experiment, participants filled out the NARQ and were debriefed at the end of the experiment.

### Behavioural data

The social competencies ratings of the photos were analysed by averaging the scores (analogue scale ranging from 0 to 100) separately for each condition. Even though the rating task was mainly used to focus the participant’s attention on the faces, we analysed those data as well, in an exploratory manner, to determine whether there were any narcissism-related effects on the ratings.

### Electrophysiological recording and pre-processing

EEG recording was similar to Ohmann et al. (2016): sixty-one scalp electrodes were set up in accordance with the international 10-20 system (FP1, FP2, F7, F3, Fz, F4, F8, FC5, FC1, FC2, FC6, T7, C3, Cz, C4, T8, CP5, CP1, CP2, CP6, P7, P3, Pz, P4, P8, FCz, O1, Oz, O2, AF7, AF3, AF4, AF8, F5, F1, F2, F6, C3’, FT7, FC3, FC4, FT8, C4’, C5, C1, C2, C6, TP7, CP3, CPz, CP4, TP8, P5, P1, P2, P6, PO7, PO3, POz, PO4, PO8; Jasper, 1958). The active Ag/AgCl electrodes (*actiCAP;* Brain Products, Germany) were referenced against the left mastoid. Horizontal and vertical electrooculograms (EOG) were derived from two electrodes located on the outer right and left canthi and from an electrode below the left eye, respectively. To record the data, *BrainAmp Vision Recorder* (Brain Products) was used. Electrode impedances were held constantly below 10 kΩ and were digitised at a sampling rate of 500 Hz via BrainAmp DC (Brain Products). EEG data were filtered online with a notch filter at line frequency (50 Hz) and a low-pass filter with a cutoff frequency at 70 Hz. Several operations were performed to analyse the recorded EEG-data offline: A high-pass filter with a cutoff selfie frequency at 0.1 Hz was applied and the EEG-data were, thereafter, divided into segments ranging from 100 ms before until 800 ms after stimulus onset. Following a baseline correction (starting 100 ms before stimulus onset), artefacts were rejected with a criterion of ±500 μV. Confounding influences resulting from eye movements were eliminated using the *Gratton & Coles* ocular correction (Gratton, Coles, & Donchin, 1983) before a second baseline correction starting 100 ms before stimulus onset was conducted. Subsequently, we applied a second artefact rejection with a stricter criterion of ±100 μV. The EEG data were averaged across the segments in each condition, and the data from all 61 electrode sites were transformed with a current source density (CSD) analysis. CSD transformed signals are reference-free and less affected by overlapping, no-process related activity (e.g., Luck, 2014). For all three conditions (Self, Celebrity, Stranger), grand averages were calculated. In the Self condition, 1,507 segments were used in total to calculate the grand average waveform; the average number of segments per participant was *M* = 25.54 (*SD* = 5.83). In the Celebrity condition, we could draw on 1,535 segments (*M* = 26.02, *SD* = 5.76), and in the Stranger condition, we also used 1,535 segments (*M* = 26.02, *SD* = 5.29).

### Electrophysiological data analysis

In previous research, P1 and N170 amplitudes were derived from posterior electrode sites from both hemispheres, including the channels P7/8 and PO7/8 (Brown, El-Deredy, & Blanchette, 2010; Keyes et al., 2010; Rellecke, Sommer, & Schacht, 2012). In the current study, the topographical distribution of the neural activity indicated by CSD-maps of the grand averages suggested the same localization of the ERP components (see Figure 2). We analysed peak amplitudes of the ERP components as done in other ERP studies (see for example Brown et al., 2010; Ratner & Amodio, 2013). We focused on the same time windows as Ohmann et al. (2016) for inspecting P1 (80–120 ms after stimulus onset) and N170 (120–220 ms after stimulus onset). We averaged peak amplitudes across the abovementioned channels of each hemisphere and chose those electrode sites for further statistical analyses at which the ERP components of interest were maximal (Ohmann et al., 2016). This implies that we derived the P1 component from the channels P8/PO8 and the N170 component from P7/PO7. Due to technical noise at the electrode site PO7 for three participants, the averaged EEG-signal at PO7/P7 was slightly noisier than the signal at PO8/P8 (Figure 2). Because the data of these three participants showed a clear P1 and N170 component at PO7 and on behalf of good practice, we did not exclude these data.

### Statistical analyses

The three dependent variables of interest (the social competencies rating as well as the P1 and N170 components) were separately analysed with the within-subject factor Face Type (Self, Celebrity, Stranger). To account for the nested structure of the data (i.e., three conditions within each participant), we used multilevel modelling (Baayen, Davidson, & Bates, 2008). Multilevel models are extensions of common regression analyses that respect dependency among data (i.e., dependency due to within-subjects designs). In addition, multilevel models also allow individual differences in the dependent variables to be considered rather than averaging across participants—here individual differences in social competencies ratings and both ERP components. Thus, participants were included as random effects variable; that is, intercepts in the dependent variable(s) were allowed to vary between participants. To estimate model parameters we used maximum likelihood estimation (Twisk, 2006). Allowing intercepts to vary, in comparison to keeping intercepts fixed, improved the model fit for the multilevel models testing the P1 component, *SD* = 12.21 (95% CI: 10.46, 15.56), χ^2^ (1) = 110.47, *p* < 0.001, and the N170 component, *SD* = 11.57 (95% CI: 9.52, 14.03), χ^2^ (1) = 131.92, *p* < 0.001.

To test the general effect of Face Type on the dependent variable(s), two dummy variables were entered as predictors (fixed effects). As we were mainly interested in the Self condition, the first dummy variable represented the differences in the dependent variables between the Self and the Celebrity conditions; the second dummy variable referred to the differences between the Self and the Stranger conditions. In a second step, we included both NARQ subscales (Admiration and Rivalry*)* as continuous predictor variables as well as all possible interaction terms of the predictors in the models. Treating Admiration and Rivalry as continuous variables—instead of dichotomizing them via median split—preserved individual-level variation and allowed us to predict along the continuum of these variables (Rucker, McShane, & Preacher, 2015). Admiration and Rivalry scores were centred as recommended by Aiken and West (1991). The analyses were run with *R* by applying the *R*-package nlme (Pinheiro, Bates, DebRoy, Sarkar, & R Development Core Team, 2010). To detail significant interaction effects, follow-up simple slope analyses were performed (Bauer & Curran, 2005). Moreover, the Johnson-Neyman (J-N) technique was used to calculate the regions of significance concerning the interaction effects observed (Johnson & Fay, 1950; Johnson & Neyman, 1936).

## Results

### Social competencies rating

The multilevel model for Face Type showed that participants ascribed significantly higher social competencies when viewing a celebrity’s face (mean ± standard error: 51.70 ± 4.86) compared with their own face (49.70 ± 5.41), *b* = 2.00, *t*(116) = 2.33, *p* = 0.022. The difference in the social competencies rating between Self and Stranger (50.93 ± 4.38) was not significant, *b* = 1.23, *t*(116) = 1.43, *p* = 0.157. In the next step, both NARQ subscales as well as every possible interaction term were entered into the model; the results of this model are presented in Table 1. In addition to the main effect of Face Type, we found a significant main effect of Admiration, a significant Admiration by Rivalry interaction and a significant interaction effect between the Self-Stranger dummy variable and the Rivalry subscale.

**Table 1:** Parameter estimates for the multilevel model analysing effects on the social competencies rating

	*b*	SE *b*	95% CI	*p*
Intercept	50.07	0.64	48.85, 51.29	<0.001
Self vs. Celebrity	1.87	0.86	0.22, 3.53	0.032
Self vs. Stranger	0.93	0.86	−0.73, 2.58	0.286
Admiration	−3.53	10.43	−5.55, −1.51	0.001
Rivalry	1.74	0.90	0.01, 5.46	0.059
Admiration x Rivalry	−3.65	16.89	−6.92, −0.38	0.035
Self vs. Celebrity x Admiration	2.77	14.08	0.07, 5.46	0.052
Self vs. Stranger x Admiration	3.87	14.09	1.17, 6.56	0.007
Self vs. Celebrity x Rivalry	−1.94	12.17	-4.27, 0.39	0.114
Self vs. Stranger x Rivalry	−3.47	12.17	−5.80, −1.14	0.005
Self vs. Celebrity x Admiration x Rivalry	1.23	22.82	−3.14, 5.60	0.591
Self vs. Stranger x Admiration x Rivalry	2.96	22.82	−1.40, 7.33	0.197

SE = standard error; CI = confidence interval.

**Figure 1.**
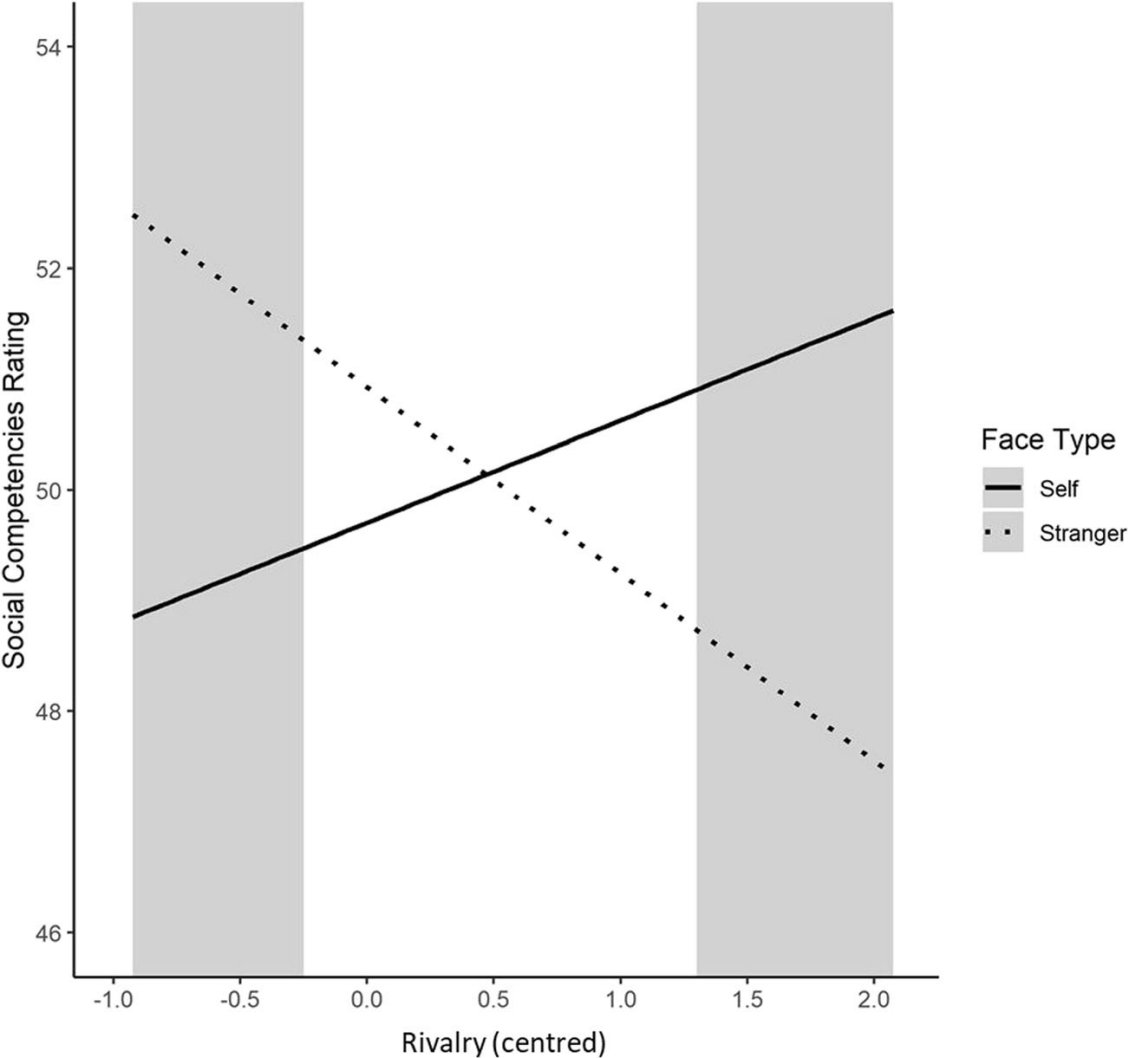
Interaction effect of Rivalry with face type (Self, Stranger) on the social competencies rating. The analogue scale of the social competencies rating ranged from 0 to 100. Grey areas indicate the regions of significance of this interaction effect. The interaction effect is illustrated (only) for the range of the observed centred Rivalry scores in our study.

To further investigate the significant interaction effect (Self vs. Stranger x Rivalry), a simple slope analysis was conducted with Rivalry scores being fixed one standard deviation above and below the mean (Preacher, Curran, & Bauer, 2006). The analysis revealed that when Rivalry scores are fixed one standard deviation below the mean, one’s own face is ascribed significantly lower social competencies than a stranger’s face; *b* = 3.39 (*SE* = 1.16), *z* = 2.91, *p* = 0.004. For Rivalry scores one standard deviation above mean, the higher social competencies rating for one’s own face compared to a stranger’s face was not significant, *b* = −1.53 (*SE* = 1.19), *z* = 1.28, *p* = 0.199. The J-N technique indicated a significant interaction effect for centred Rivalry scores < −0.25 and > 1.30 (Figure 1).

### ERP data

**Figure 2.**
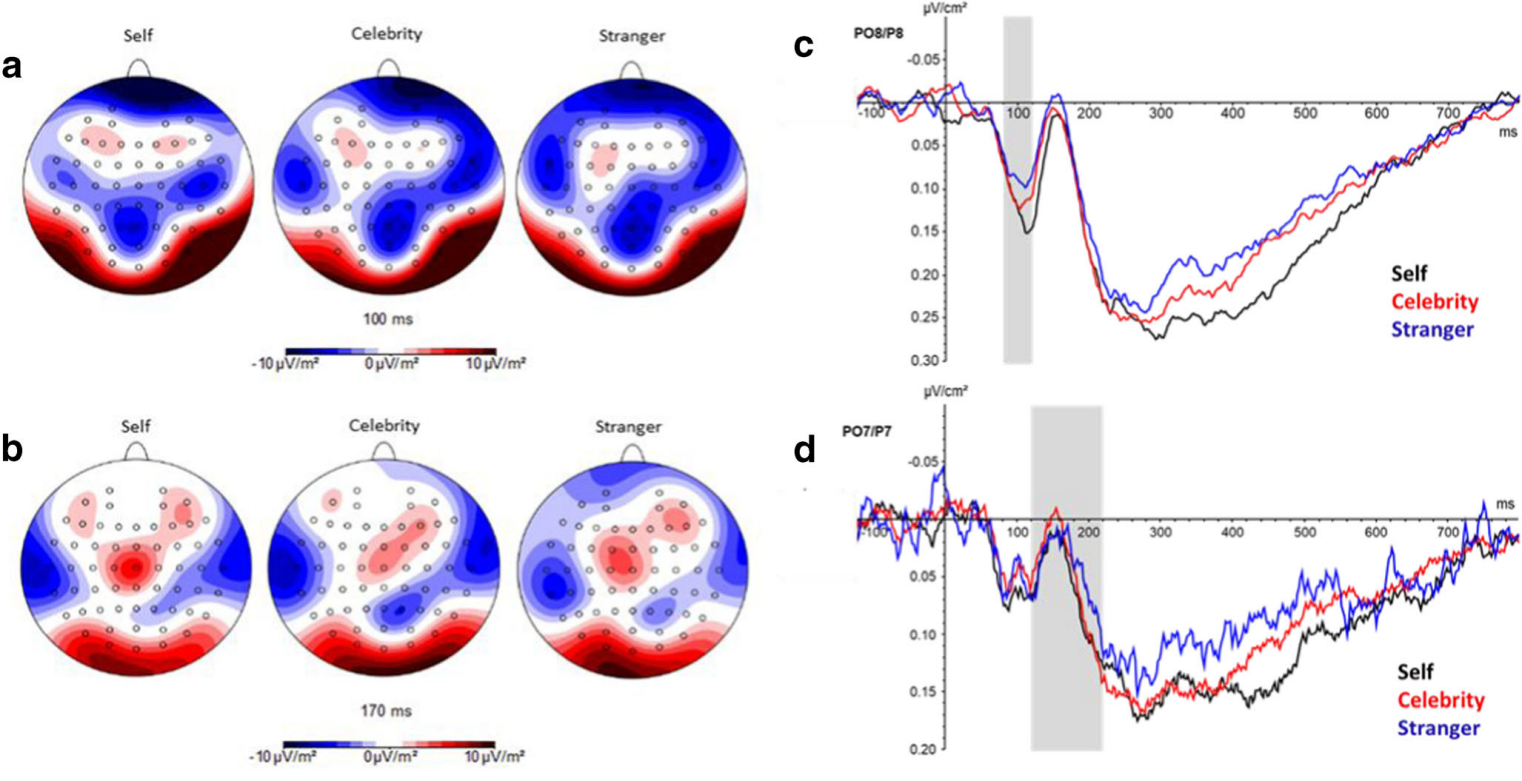
Event-related potentials. Topographic maps of mean CSD-transformed ERPs for the three conditions (Self, Celebrity, Stranger) at (**A**) 100 ms and (**B**) 170 ms after stimulus presentation. (**C**) Grand average CSD-ERP waveforms that were averaged across electrode sites PO8 and P8 for all three conditions. The grey area indicates the time window that was used to inspect the P1 amplitude. (**D**) Grand average CSD-ERP waveforms that were averaged across electrode sites PO7 and P7 for all three conditions. The grey area shows the time window that was used to inspect the N170 amplitude.

Topographic maps of the CSD-transformed ERPs indicated enhanced right hemispheric neural activity in the time interval of the P1 component (Figure 2A) and enhanced left hemispheric neural activity in the time interval of the N170 component (Figure 2B). Figure 2C shows grand average CSD-ERP waveforms for all three conditions that were averaged across the right parieto-occipital (PO8) and the right parietal electrode site (P8), where a pronounced P1 component is shown. The N170 component is presented in Figure 2D, which shows grand average CSD-ERP waveforms for all three conditions that were averaged across the left parieto-occipital (PO7) and the left parietal electrode site (P7).

#### P1 component and NARQ subscales

The multilevel model testing for the general effect of Face Type effects on the P1 amplitude (i.e., without the subscales included) did neither reveal a significant difference between Self (mean ± standard error: 0.187 ± 0.020 μV/cm^2^) and Celebrity (0.200 ± 0.020 μV/cm^2^), *b* = 0.012, *t*(116) = 0.89, *p* = 0.370, nor between Self and Stranger (0.180 ± 0.020 μV/cm^2^), *b* = -0.007, *t*(116) = −0.51, *p* = 0.610. Including the NARQ subscales and all possible interaction terms in the model led to the results presented in Table 2.

**Table 2.** Parameter estimates for the multilevel model analysing effects on P1 (μV/cm^2^)

	*b*	SE *b*	95% CI	*p*
Intercept	0.182	0.020	0.143, 0.221	<0.001
Self vs. Celebrity	0.019	0.014	−0.007, 0.046	0.171
Self vs. Stranger	−0.002	0.014	−0.028, 0.025	0.896
Admiration	−0.063	0.033	−0.127, 0.001	0.063
Rivalry	0.047	0.029	−0.009, 0.102	0.110
Admiration x Rivalry	0.049	0.054	−0.055, 0.153	0.369
Self vs. Celebrity x Admiration	0.045	0.023	0.002, 0.089	0.047
Self vs. Stranger x Admiration	0.026	0.023	−0.017, 0.070	0.246
Self vs. Celebrity x Rivalry	−0.040	0.020	−0.077, −0.002	0.046
Self vs. Stranger x Rivalry	−0.004	0.020	−0.041, 0.034	0.858
Self vs. Celebrity x Admiration x Rivalry	−0.067	0.037	−0.137, 0.003	0.072
Self vs. Stranger x Admiration x Rivalry	−0.052	0.037	−0.122, 0.018	0.157

SE = standard error; CI = confidence interval.

**Figure 3.**
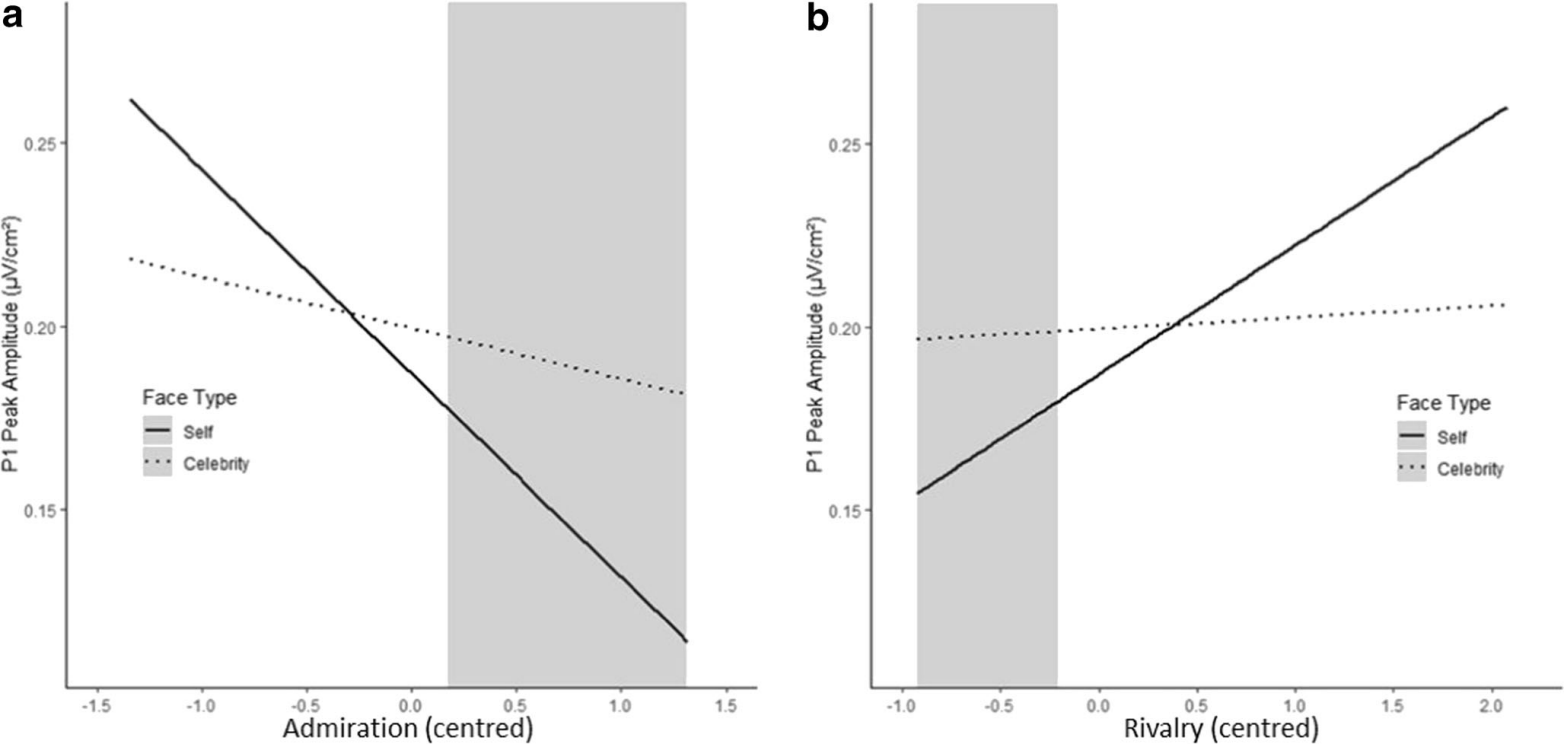
Interaction effects of (A) Admiration and (B) Rivalry with face type (Self, Celebrity) on P1. Grey areas indicate the regions of significance of these interaction effects. The interaction effects are illustrated (only) for the range of the observed centred Admiration and Rivalry scores in our study.

Most importantly, differences in the P1 amplitude between Self and Celebrity were moderated by both NARQ subscales (Admiration and Rivalry). Interaction effects were probed as previously described. The simple slope analysis of the Self vs. Celebrity by Admiration interaction showed that when Admiration is one standard deviation above the mean, the P1 is significantly smaller when observing one’s own face compared to a celebrity’s face; *b* = 0.051 (*SE* = 0.020), *z* = 2.57, *p* = 0.010. When looking at one standard deviation below mean, the effect of the condition was not significant; *b* = −0.013 (*SE* = 0.021), *z* = −0.63, *p* = 0.530. This finding is again corroborated by the J-N technique and the region of significance for high Admiration starts at centered Admiration scores of 0.18 (Figure 3A).

The simple slope analysis of the Self vs. Celebrity by Rivalry interaction revealed that, when Rivalry scores were held constant one standard deviation below the mean, P1 is significantly higher when viewing a celebrity’s face compared with when viewing one’s own face, *b* = 0.047 (*SE* = 0.019), *z* = 2.52, *p* = 0.012. The difference in P1 amplitude for viewing a celebrity compared with one’s own face when Rivalry scores were fixed one standard deviation above mean was not significant, *b* = −0.009 (*SE* = 0.019), *z* = 0.46, *p* = 0.643. The J-N technique indicated a significant interaction effect for centred Rivalry scores below −0.21 (Figure 3B).

#### N170 component and NARQ subscales

The multilevel model for the general effect of Face Type on the N170 amplitude (i.e. without the NARQ subscales) indicated a significantly higher N170 peak amplitude for Self (−0.107 ± 0.019 μV/cm^2^) than for Celebrity (−0.084 ± 0.018 μV/cm^2^), *b* = 0.023, *t*(116) = 2.11, *p* = 0.037. We also found a higher N170 peak amplitude in the Self than in the Stranger condition (−0.074 ± 0.016 μV/cm^2^), *b* = 0.033, *t*(116) = 3.10, *p* = 0.002. Note that beta-coefficients are positive as the N170 represents a negative deflection in the ERP. Including the NARQ subscales and every possible interaction term led to the results presented in Table 3. Most importantly, we found that the Self-Stranger difference in the N170 amplitude was moderated by Rivalry.

**Table 3.** Parameter estimates for the multilevel model analysing effects on N170 (μV/cm^2^)

	*b*	SE *b*	95% CI	*P*
Intercept	−0.108	0.018	−0.142, −0.073	<0.001
Self vs. Celebrity	0.025	0.011	0.004, 0.046	0.023
Self vs. Stranger	0.038	0.011	0.017, 0.059	<0.001
Admiration	0.045	0.029	−0.011, 0.010	0.127
Rivalry	−0.031	0.025	−0.080, 0.018	0.230
Admiration*Rivalry	0.008	0.047	−0.084, 0.0100	0.863
Self vs. Celebrity*Admiration	0.020	0.018	−0.014, 0.055	0.261
Self vs. Stranger*Admiration	−0.005	0.018	−0.039, 0.030	0.795
Self vs. Celebrity*Rivalry	0.018	0.016	−0.012, 0.048	0.245
Self vs. Stranger*Rivalry	0.031	0.016	0.002, 0.061	0.046
Self vs. Celebrity*Admiration*Rivalry	−0.026	0.029	−0.082, 0.030	0.367
Self vs. Stranger*Admiration*Rivalry	−0.049	0.029	-0.104, 0.007	0.096

SE = standard error; CI = confidence interval.

**Figure 4.**
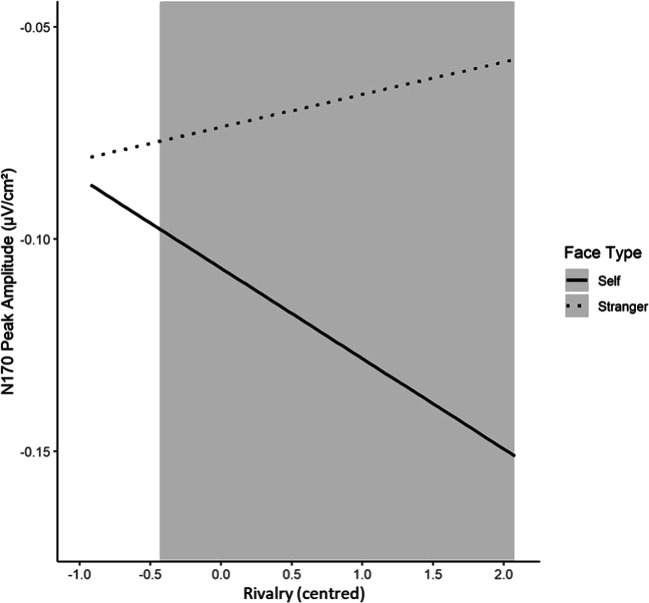
Interaction effect of Rivalry with face type (Self, Stranger) on N170. The grey area indicates the regions of significance of this interaction effect. The interaction effect is illustrated (only) for the range of the observed centred Rivalry scores in our study. N170 represents a negative deflection in the ERP.

A simple slope analysis was conducted to probe for the interaction. For Rivalry scores held constant 1 standard deviation above the mean, the results indicate a significant reduction of the N170 peak amplitude when observing one’s own face compared with viewing a stranger’s face, *b* = 0.060 (*SE* = 0.015), *z* = 3.97, *p* < 0.001. For one standard deviation below the mean, the difference between both conditions was not significant, *b* = 0.016 (*SE* = 0.015), *z* = 1.08, *p* < 0.279. The J-N technique indicated a significant interaction effect for centralised Rivalry scores > −0.43 (Figure 4).

## Discussion

We found that Admiration and Rivalry, two narcissism dimensions (Back et al., 2013), varied with neural correlates of face processing, within the first 200 ms after stimulus onset. By showing participants their own face, a celebrity’s face, and a stranger’s face, we discovered moderating effects of Admiration and Rivalry on two ERP components: The P1 component covaried with Admiration while both the P1 and the N170 component covaried with Rivalry. The results only partly reflected our assumptions that P1 and N170 varied for participants high in Admiration when viewing one´s own face and for participants high in Rivalry when viewing a stranger´s face. We discuss possible mechanisms underlying the observed effects below.

### Narcissism and very early face processing reflected in P1

Results for P1 showed that both Admiration and Rivalry moderated the effect of the Self-Celebrity comparison. Viewing one’s own face compared with a stranger’s face, however, was neither influenced by Admiration nor Rivalry. Thus, the following discussion about possible influences of Admiration and Rivalry on P1 focuses on the Self-Celebrity comparison.

#### Admiration and attentional inhibition of one’s own face

We mentioned earlier that the P1 was interpreted as an indicator of attentional selection processes determining which information enters (and does not enter) consciousness for further stimulus processing (Railo et al., 2011). Hereafter, it could be reasoned that the lower P1 for participants with high Admiration when viewing themselves compared to a celebrity reflects the inhibition of attention to their own face. This seems to be a paradox at first sight: One could argue that individuals high in narcissism process photos of their face even more intensely because of the exaggerated positive self-views they report (Campbell, Rudich, & Sedikides, 2002) and the joy of looking at themselves, as stated in the Narcissistic Personality Inventory (Raskin & Terry, 1988). However, one also could argue that individuals with high Admiration scores avoid processing their own face to protect their explicit grandiosity against potentially contradicting information—meaning protecting their grandiosity against the perception of an “imperfect” photo. According to the consistency theory of psychological functioning (Grawe, 2004; Grawe, 2007), the human organism has developed control mechanisms to coordinate simultaneously ongoing processes to ensure their consistency (Rumelhart & McClelland, 1986). Inconsistency of processes—reflected for example in conscious or unconscious motivational conflict—impairs mental functioning, leads to distress, and may in the long-term cause mental disorders and even suicidal tendencies (Grawe, 2004). Thus, attentional inhibition can be regarded as one mechanism to ensure consistency of psychological functioning (Grawe, 2004; Diamond, 2013). When, for example, a particular motivational goal is activated (such as the goal to feel grandiose in people with high Admiration), any cognitions, emotions, and (in this case) perceptions that interfere with this goal are inhibited to ensure consistency and, thereby, goal-directed behaviour (Grawe, 2007). Accordingly, the lower P1 might reflect a mechanism related to narcissism that serves the protection of one’s grandiosity against inconsistent perceptions, that is, a potentially “imperfect” picture.

Of course, a photo of one’s own face does not have to be ego-threatening but could rather provide evidence for one’s attractiveness; however, considering the overarching motivational goal of experiencing grandiosity in narcissism (Morf, Torchetti, & Schürch, 2011; Back et al., 2013), the spontaneous, unadorned view of one’s own face may actually fall behind that goal. That is, the perceptual input (photo) is inconsistent with the exaggerated grandiose cognitions and the overestimated self-view of their attractiveness (Gabriel, Critelli, & Ee, 1994). Moreover, some research suggested that the overtly, explicitly stated high self-view of people with high narcissism scores is actually accompanied by a rather low implicit self-view (Zeigler-Hill & Jordan, 2011; Zeigler-Hill, 2006). Thus, individuals with high narcissism scores seem not necessarily to experience grandiosity or perfectness at all levels; there seem to be aspects of themselves that they may not fully endorse or that they experience as falling behind their grandiose standards. Thus, the lower P1 might reflect attentional inhibition to shield the explicit grandiosity against this implicit vulnerability (Morf et al., 2011; Horvath & Morf, 2009).

#### Admiration and expectancy-driven self-perception

Besides attentional inhibition, the influence of grandiose fantasies (associated with Admiration; Back et al., 2013) on perception also could explain the alterations in P1. These cognitive structures could be connected to an *expectancy-driven* perception of one’s face, while *stimulus-driven processing* of the actual photo might be tuned down (Gilbert & Li, 2013; Engel, Fries, & Singer, 2001). More precisely, perceptions of one’s face might be driven by expectations about one’s attractive looks instead of the actual sensory input. As a consequence, fewer neural resources might be mobilised for processing the sensory input, which could result in a lower P1. In line with this, several researchers suggested that perception is not only a passive and stimulus-driven but foremost a constructive and expectancy-driven process in which existing cognitive structures can highly modulate the processing of sensory input (Gilbert & Li, 2013; Engel et al., 2001; Damasio, 1990; Edelman, 1989). Interestingly, P1 was shown to vary with low-level information of visual stimuli (Rossion & Jacques, 2008), and it is tempting, although speculative, to say that the lower P1, which was observed for high Admiration scores in the Self condition, reflected the reduced processing of the low-level features of one’s face.

If cognitive structures representing one’s grandiosity modulate early information processing, this might account for example for the findings that highly narcissistic people overestimate their attractiveness (Gabriel et al., 1994) and their performance (Campbell, Goodie, & Foster, 2004). These findings might be a result of highly expectancy-driven self-perception, whereas processing of actual sensory input from self-relevant stimuli might habitually be reduced in narcissism. Obviously, this interpretation does not contradict but rather complement the attentional inhibition hypotheses. While the attentional inhibition hypothesis emphasises that one’s grandiosity needs to be protected (against inconsistent self-relevant information), this interpretation accentuates the fact that expectations of grandiosity highly influence the perceptional process. Both mechanisms can co-occur and, in either way, narcissistic grandiosity is stabilised.

#### Admiration and attentional facilitation to people of high social status

The previously discussed hypotheses concentrated on the processing of self-photos. At the same time, however, there could have been intensified processing of the celebrity’s face, which also might have contributed to the P1 difference between Self and Celebrity. Participants with high Admiration scores might have paid special attention to the Celebrity photos, leading to a higher P1. Campbell and Green (2007) postulated that highly narcissistic people tend to affiliate with people with a high social status, which serves to stabilise one’s own grandiosity. Consequently, people exhibiting high social status can be socially highly relevant to narcissistic individuals, and it was shown that the social relevance of another person leads to intensified face processing, which is reflected in an enlarged P1 (Bublatzky, Gerdes, White, Riemer, & Alpers, 2014). Thus, the relatively high P1 amplitude in participants with high Admiration in the Celebrity condition, compared with the Self condition, might reflect the assignment of social relevance to famous people.

#### Rivalry and comparison with people of high social status

Although the two NARC subscales are positively correlated (Back et al., 2013), we found a quite different pattern for P1 and Rivalry. Interestingly, participants with *low* Rivalry showed a significantly smaller P1 amplitude when observing their own face compared with a celebrity’s face. High Rivalry participants did not show a significant P1 difference between viewing one’s own face and that of a celebrity. Back et al. (2013) suggested that the mechanism of Rivalry incorporates the tendency to compare oneself with perceived social rivals. Consequently, not only the celebrity’s but also one’s own face should be important stimuli for high Rivalry participants, leading to more intense processing of both stimuli. This might be reflected in the relatively high and not significantly different P1 in the Self and Celebrity condition. In contrast, for participants with low Rivalry scores, a picture of one’s face might be less emotionally salient compared with a celebrity’s face, leading to a lower P1 amplitude (Vuilleumier, 2005).

Rivalry moderated the effect of the Self-Celebrity comparison on the P1 amplitude in an opposite way than Admiration. This finding is interesting considering the high positive correlation of both subscales (*r* = 0.61; Back et al., 2013). The opposite effect of Admiration and Rivalry on very early face processing supports that both dimensions should be distinguished as different pathways that enable highly narcissistic people to stabilise their grandiose self-view (Back et al., 2013).

### Rivalry and structural encoding of one’s own face indicated by N170

With regard to the N170 amplitude, reflecting higher-order face-sensitive perceptual processes at a later processing stage (Rossion & Jacques, 2011), we successfully replicated the so-called self-effect (Keyes et al., 2010); i.e., enhanced N170 amplitudes for one’s own face compared with a celebrity’s and a stranger’s face. Also, we found a moderation effect of Rivalry on the Self-Stranger difference. The difference between Self and Stranger (higher N170 in the Self condition) was larger for higher Rivalry scores. Only for participants with very low scores in Rivalry, there was no significant Self-Stranger difference.

As stated above, Ofan et al. (2014) demonstrated that the N170 is higher when participants are afraid of showing racial prejudices. The authors argued that social anxiety could facilitate visual face processing activity, indicated by a higher N170, to promote adequate response strategies for preventing social disapproval because of showing racial prejudices. Transferred to our findings, a higher N170 in the Self (than in the Stranger) condition for increasing Rivalry scores also might reflect the mobilisation of defensive response strategies. In contrast to Admiration, Rivalry is accompanied by massive efforts to protect one’s grandiosity from real and imagined attacks and with constant fears of ego-threats (Back et al., 2013). When the own face of an individual scoring high in Rivalry becomes the focus of attention, this self-protection strategy might be activated, leading to enhanced stimulus processing of their own face—as reflected in a higher N170. This might enable the individual to prepare for potentially ego-threatening feedback and to initiate self-protective and aggressive behaviour if necessary—as Back et al. (2013) postulated for the mechanism of Rivalry. Thus, whereas Admiration might be connected to the reduced processing of one’s own face at an early face processing stage (lower P1) to possibly protect one’s grandiosity, Rivalry might be associated with a facilitated face processing at a later stage (enhanced N170), which could reflect the mobilisation of defensive systems.

### Rivalry and the social competencies rating: devaluation of strangers

In addition to ERP components, we investigated the variations of Admiration and Rivalry with the social competencies rating. In general, participants gave higher social competencies ratings for celebrities than for themselves. Considering the narcissism scales, we found that Rivalry showed a moderating effect on the Self-Stranger comparison: Participants high in Rivalry ascribed higher social competencies to their own face than to the stranger´s face. In contrast, participants low in Rivalry ascribed more social competencies to strangers and attributed less to themselves. This is consistent with the postulation that Rivalry, as a strategy to maintain narcissistic grandiosity, is associated with the devaluation of other people and striving for supremacy (Back et al., 2013). Thereby, the social competencies rating pointed to the ecological validity of the Rivalry scale.

### Limitations and Future Research

Unfortunately, it was not possible to create a baseline condition that would have allowed for a comparison with Self, Celebrity, and Stranger. With regard to narcissism, every kind of face is possibly connected to unique alterations in sensory processing because of the broad influence of narcissism on self- and other-perception (Morf et al., 2011). Thus, it was only possible to compare the different face conditions with each other. Consequently, the interactions between face-comparisons and the narcissism dimensions had to be interpreted from the viewpoint of both face conditions involved in the respective comparison. Therefore, in the future, we need to create a (more) neutral condition (e.g., an object) for comparison. Furthermore, it is still difficult to interpret the meaning of larger amplitudes of a component: an increase could mean either more intense processing or stronger inhibition. Thus, a further systematic investigation of the neural correlates and their personality-related variations is inevitable. Moreover, because we only chose one well-known female and male celebrity in the current study, we cannot rule out that the observed pattern cannot be generalised to all famous people. Therefore, other celebrities and other people with high social status could be used in future research. Finally, we want to discuss the sample size in the current study. There is no general convention concerning power analyses in the multilevel approach given the variety of different multilevel models. With simulations, Maas and Hox (2005) demonstrated that a minimum sample size of 50 at the group level is needed to ensure acceptable accuracy of parameter estimates. The current study met this criterion with a sample size of 59 at the group level. Small sample sizes at level 1 do not pose a problem by themselves (Hox, 2013). Thus, the current results constituted a promising lead we should explore in future studies.

For future research, it would be interesting to alter pictures of the participant with regard to attractiveness and to investigate whether potential effects on P1 are moderated by Admiration. This could be realised by having participants bring a favourable photo of themselves to the experiment and contrast this with an unadorned photo taken by the experimenter—of course, one would have to match these pictures according to low-level features and would have to verify subjective differences in attractiveness with a manipulation check. Whereas participants with low Admiration scores might process pictures of themselves that vary in attractiveness differently. Due to potential emotional impacts of advantageous and less advantageous photos, individuals high in Admiration might not show these variations because of the discussed phenomena concerning attentional inhibition and expectancy-driven perception. Furthermore, it would be interesting to examine whether there are similar variations of Rivalry and Admiration with P1 and N170 for other self-relevant stimuli, such as someone’s name, that also elicit P1 and N170 (Tacikowski, Jednorog, Marchewka, & Nowicka, 2011). Finally, it would be interesting to expand the current research to the investigation of other well-studied ERPs. It might be worthwhile examining whether there are associations between narcissism and ERP components occurring in error processing. Previous research demonstrated that narcissism is associated with specific reactions to failure (Kernis, & Sun, 1994; Campbell et al., 2004), which also might be based on neural variations in very early error processing.

## Conclusions

Inspired by the Greek myth of Narcissus, the starting point of our modern understanding of narcissism, we were interested in the question of whether narcissism is connected to unique alterations in face perception on a neural level. We demonstrated that two dimensions of narcissism, Admiration and Rivalry, vary in their own specific ways with two ERP components of face processing, P1 and N170. The current results exemplify that ERP research is well suited to uncover automatic neural responses in narcissism and might, in future studies, further elucidate the very nature of this complex and controversially discussed construct.
